# The Genetic Structure of Chinese Hui Ethnic Group Revealed by Complete Mitochondrial Genome Analyses Using Massively Parallel Sequencing

**DOI:** 10.3390/genes11111352

**Published:** 2020-11-14

**Authors:** Chong Chen, Yuchun Li, Ruiyang Tao, Xiaoye Jin, Yuxin Guo, Wei Cui, Anqi Chen, Yue Yang, Xingru Zhang, Jingyi Zhang, Chengtao Li, Bofeng Zhu

**Affiliations:** 1 Key Laboratory of Shaanxi Province for Craniofacial Precision Medicine Research, College of Stomatology, Xi’an Jiaotong University, Xi’an 710004, China; cc18883368974@stu.xjtu.edu.cn (C.C.); jinxy0901@stu.xjtu.edu.cn (X.J.); guoyuxin_004@stu.xjtu.edu.cn (Y.G.); olliecheung@stu.xjtu.edu.cn (X.Z.); 2 Shanghai Key Laboratory of Forensic Medicine, Shanghai Forensic Service Platform, Academy of Forensic Science, Ministry of Justice, Shanghai 200063, China; 2017324010020@stu.scu.edu.cn (R.T.); 19111010078@fudan.edu.cn (A.C.); Yangyue110173@163.com (Y.Y.); 20174221083@stu.suda.edu.cn (J.Z.); 3 Multi-Omics Innovative Research Center of Forensic Identification, Department of Forensic Genetics, School of Forensic Medicine, Southern Medical University, Guangzhou 510515, China; cuiwei3702@stu.xjtu.edu.cn; 4 State Key Laboratory of Genetic Resources and Evolution/Key Laboratory of Healthy Aging Research of Yunnan Province, Kunming Institute of Zoology, Chinese Academy of Sciences, Kunming 650223, China; liyuchun@mail.kiz.ac.cn; 5 Institute of Forensic Medicine, West China School of Basic Medical Sciences & Forensic Medicine, Sichuan University, Chengdu 610017, China; 6 Department of Forensic Medicine, Shanghai Medical College of Fudan University, Shanghai 200032, China; 7 School of Basic Medicine, Inner Mongolia Medical University, Hohhot 010030, China; 8 College of Forensic Medicine, Xi’an Jiaotong University Health Science Center, Xi’an 710061, China

**Keywords:** complete mitochondrial genome, mitochondrial haplogroup, massively parallel sequencing, population genetics, Hui minority

## Abstract

Mitochondrial DNA (mtDNA), coupled with maternal inheritance and relatively high mutation rates, provides a pivotal way for us to investigate the formation histories of populations. The Hui minority with Islamic faith is one of the most widely distributed ethnic groups in China. However, the exploration of Hui’s genetic architecture from the complete mitochondrial genome perspective has not been detected yet. Therefore, in this study, we employed the complete mitochondrial genomes of 98 healthy and unrelated individuals from Northwest China, as well as 99 previously published populations containing 7274 individuals from all over the world as reference data, to comprehensively dissect the matrilineal landscape of Hui group. Our results demonstrated that Hui group exhibited closer genetic relationships with Chinese Han populations from different regions, which was largely attributable to the widespread of haplogroups D4, D5, M7, B4, and F1 in these populations. The demographic expansion of Hui group might occur during the Late Pleistocene. Finally, we also found that Hui group might have gene exchanges with Uygur, Tibetan, and Tajik groups in different degrees and retain minor genetic imprint of European-specific lineages, therefore, hinting the existence of multi-ethnic integration events in shaping the genetic landscape of Chinese Hui group.

## 1. Introduction

The Hui minority is one of the largest and widespread Chinese ethnic groups with Muslim followers. Unlike many Muslim populations, such as Uygur and Kazak, the Hui people exhibit a greater degree of assimilation with the majority of Han people in terms of language and culture. According to historical documents, the ancestors of the Hui minority in China could be traced back to the Tang dynasty (618–907 AD), but it was in the Yuan dynasty (1271–1368 AD) that the Hui really started the multi-ethnic integration [1]. During the Mongolian-Yuan period, numerous Muslims from Central and Western Asia such as Persia, Arabia, and Turkic settled in China, laying a population foundation for the Hui group to eventually form an ethnic group in Ming dynasty (1368–1644 AD) [2]. The fusion of Han, Tibetan, Mongolian, and other ethnic components into the Hui group has also attracted the attention of scholars [3,4]. 

In recent reports, some genetic evidences have been employed to dissect the demographic history and genetic landscape of the Hui ethnic group. For example, the origin of Hui group was attributed to the substantial assimilation of East Asians and minor assimilation of West Eurasians based on the paternal Y chromosome analyses [5]. Meanwhile, Hui was also proved to have proximal genetic relationships with Uygur, Han, Mongolian, and Salar groups [2,3,4]. However, the existing researches anatomizing the genetic background of Hui group from the perspective of maternal inheritance are relatively limited and merely based on the partial sequences of mitochondrial DNA (mtDNA), such as the mtDNA control region [3]. Therefore, the introduction of complete mitochondrial genome analyses will undoubtedly contribute to a more comprehensive interpretation of the matrilineal genetic structure of Hui group.

In this study, we employed the complete mitochondrial genomes of 98 healthy and unrelated individuals from Hui group, Northwest China, which were sequenced by the massively parallel sequencing (MPS) technique on the HiSeq X Ten system (Illumina, San Diego, CA, USA). In order to obtain an extensive understanding of the maternal genetic structure of Hui group, we collected 7274 complete mitochondrial sequences from 99 previously published populations around the world for a comparative study. The genetic history of one population reflected by mitochondrial genome may not be exactly the same as the true history, but through the extensive study of mtDNA variations of Hui individuals, it can still obtain some insights into the genetic structure of the contemporary Hui groups and the demographic episodes they might have experienced.

## 2. Materials and Methods

### 2.1. Sample Preparation and Ethical Declaration

The whole blood samples of 98 Hui individuals without consanguineous relationships were collected from Northwest China. Written informed consents and demographic information were acquired from all the participants concerned. This study was conducted with the approval of the Ethics Committee of Xi’an Jiaotong University Health Science Center (approval number: 2019–1039; XJTULAC201) and followed the ethical principles proposed by the World Medical Association [6]. 

### 2.2. Library Construction and Sequencing of Mitochondrial Genome

The library was constructed based on the MultipSeqTm AImumiCap Panel provided by Enlighten Biotechnology Company (Shanghai, China) with the following steps. Initially, the target DNA regions were acquired by multiplex PCR amplification, which contained a 5 μL RealCapChrMT mix, 10 μL 3×EnzymeHF, 1 μL template DNA (5 ng/μL), and 14 μL nuclease-free H_2_O in a volume of 30 μL system. The PCR cycling reaction was carried out as: 98 °C for 3 min; 13 cycles including 98 °C for 20 s, 58 °C for 4 min; seven cycles including 98 °C for 20 s, 72 °C for 1 min; then, 72 °C for 2 min and holding at 10 °C. The amplified products were further purified by magnetic beads. Secondly, the indexes were appended to the purified products by reamplification and the target DNA regions were enriched as well. The index-adding reaction was performed in a 30 μL system, containing 18 μL purified PCR products, 10 μL 3×EnzymeHF, 1 μL I5MRBar, and 1 μL I7MRBar. The corresponding PCR procedure was 98 °C for 2 min, then six cycles with 98 °C for 15 s, 58 °C for 15 s, and 72 °C for 15 s, lastly 72 °C for 2 min, and stop reaction in 10 °C. The reamplified products were purified again utilizing magnetic beads. Finally, the constructed library was quantified by the Qubit 2.0 Fluorometer platform (Thermo Fisher Scientific, San Jose, CA, USA) with the Qubit dsDNA HS Assay Kit (Thermo Fisher Scientific, CA, USA). The agarose gel electrophoresis was carried out to evaluate the quality of constructed library. After quantification, the library was implemented for paired-end sequencing on an Illumina HiSeq X Ten instrument.

### 2.3. Sequencing Data Analyses 

The original image data obtained from high-throughput sequencing were automatically analyzed by base recognition and converted into the original sequences in FASTQ format. To ensure the accuracy of subsequent analyses, redundant primers and indexes in initial FASTQ files were removed by the Cutadapt software (http://cutadapt.readthedocs.org/), and low-quality reads were filtered by the Trimmonmatic software (http://www.usadellab.org/cms/index.php?page=trimmomatic/). The final cleaned data were mapped to the revised Cambridge Reference Sequence [7] plus 64 bp (rCRS + 64 bp) using the Burrows-Wheeler Aligner (BWA, http://bio-bwa.sourceforge.net/) to generate the binary alignment/map (BAM) file. In order to escape false positives from contaminations of nuclear mitochondrial DNA (NUMTs ), the sequences were also compared with the human reference genome hg19 [8]. The reads successfully mapped to hg19 were extracted by the Bedtools software (https://bedtools.readthedocs.io/en/latest/) and realigned to rCRS + 64 bp to produce new BAM files using the Bowtie2 software (http://bowtie-bio.sourceforge.net/bowtie2/). Finally, the variation sites in the BAM file were identified by SAMtools v1.8 (http://samtools.sourceforge.net/) and VarScan v2.4.0 (http://varscan.sourceforge.net/), which were stored as a variant call format (VCF) file. The final consistent sequences were yielded by BCFtools v1.9 (https://samtools.github.io/bcftools/) in the FASTA file.

### 2.4. The Worldwide Populations for a Comparison Study with Hui Group

In order to explore the maternal genetic structure of Hui ethnic group more comprehensively, we screened 99 reference populations worldwide, with more than 18 individuals in each population. The detailed information and cited references of the populations were listed in Supplementary Table S1. It was worth mentioning that the sample sizes of Mongolian, Altaian-Kizhi, Buryat, and Khamnigan groups were relatively small in their respective studies. Therefore, we aggregated the complete mitogenome data of these four ethnic groups from several previous studies, respectively (Supplementary Table S1). 

### 2.5. MtDNA Haplogroup Allocation

Quality control was carried out according to the previous study [9]. The haplogroups of complete mtDNA sequences from Hui group were allocated using HaploGrep 2 (https://haplogrep.i-med.ac.at) based on PhyloTree build 17 (http://www.phylotree.org/index.htm). The haplogroups of all mtDNA sequences were also rechecked and aligned manually by Snapgene (https://www.snapgene.com/), and compared with the rCRS [7]. The final haplogroup status of the mtDNA sequencing data from 98 Hui individuals was listed in Supplementary Table S2.

### 2.6. Statistical Analyses

All the haplogroup frequencies utilized in this study were generated from complete mitochondrial sequences and calculated by the direct counting method. Statistical indexes, including haplotype diversity (Hd), nucleotide diversity (pi), number of segregating sites (s), and average number of pairwise nucleotide differences (k) were estimated using DnaSP v5 [10] based on the complete mitogenome data. Mismatch distributions with model test statistics (the sum of squared deviations, SSD; Harpending’s raggedness index, HRI), neutrality tests (Tajima’s *D* and Fu’s *Fs* tests), and analysis of molecular variance (AMOVA) were evaluated by Arlequin 3.5.1.3 [11,12] using complete mitochondrial sequences. The genetic distances (*F*-statistics, *F_ST_*) between Hui group and reference populations were also calculated by Arlequin 3.5.1.3 [11,12] based on the haplogroup frequencies. The *R* statistical package (https://www.r-project.org/) was implemented to plot the circle histogram for the pairwise *F_ST_* values between Hui and reference populations. The principal component analysis (PCA) of all populations was plotted based on the mitochondrial haplogroup frequencies using the Statistical Package for the Social Sciences (SPSS) 16.0 software. We further generated Bayesian skyline plots (BSP) from BEAST 1.6.1 [13] for Hui group and Han populations from different regions. In the BSP analysis, a strict molecular clock was selected with the fixed rate of 1.665E-8 substitutions per site per year [14]. Each Markov chain Monte Carlo (MCMC) simulation was run for 10,000,000 generations with the first 1‰ discarded as burn-in. The MCMC simulation was sampled every 1000 generations. The TN93 and Gamma + Invariant Sites model were selected. And the results were visualized with Tracer v1.4 [15]. The time of population expansion was calculated according to the previous study [16,17]. The median networks for all the haplogroups emerged in the Hui group based on 1578 worldwide individuals were constructed by Network v5.0 (https://www.fluxus-engineering.com/), and the plots were subsequently visualized in the Network Publisher (http://www.fluxus-engineering.com/index.htm).

## 3. Results and Discussion

### 3.1. Sequencing Depth of Complete Mitogenome

A total of 98 individuals, including 52 females and 46 males, were successfully sequenced on the Illumina HiSeq X Ten system. The average read depth was 3280× ± 1033× (mean ± SD) per individual. The sequencing depth for all individuals were portrayed in a box plot. As shown in Supplementary Figure S1, the mean read depth for all individuals roughly ranged from 549× to 8155× with a relatively high confidence. As a result, the MultipSeqTm AImumiCap Panel (Enlighten Biotechnology Company, Shanghai, China) could perform well in the complete mitogenome sequencing.

### 3.2. Mitochondrial Haplogroups of Hui Ethnic Group

**Figure 1:**
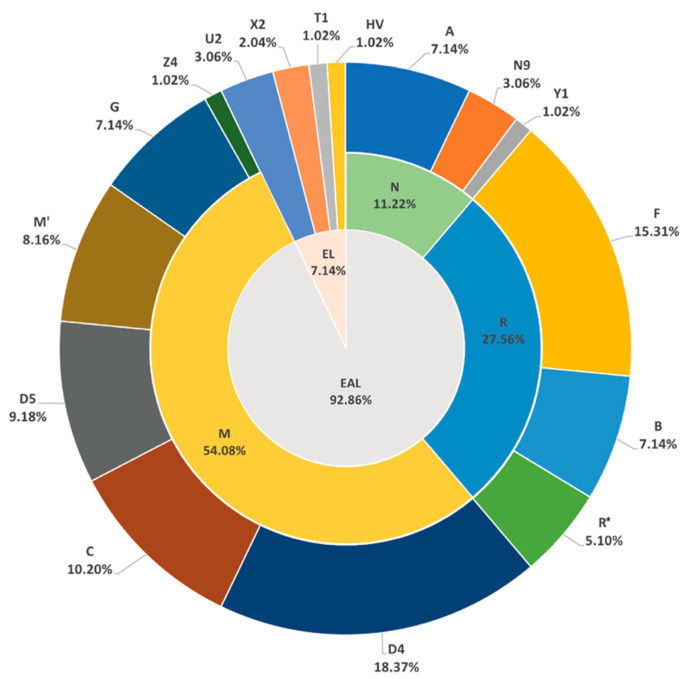
The mitochondrial haplogroup distributions of 98 Hui individuals from Northwest China. European-specific lineages represented by EL, and East Asian-specific lineages represented by EAL.

As shown in Supplementary Table S2, a total of 83 haplogroups were discerned from 98 complete mitogenomes of Hui group according to HaploGrep 2 (https://haplogrep.i-med.ac.at) based on PhyloTree build 17 (http://www.phylotree.org/index.htm) and rechecked manually. According to the previous studies, the European-specific lineages commonly encompassed haplogroups H, HV, R0, R1, R2, JT, U, N1, N2, W, and X [18,19]. Whereas, the East Asian-specific lineages were reported to contain haplogroups C, D, G, Z, E, M9a, M7, M13, A, B, F, R9c, Y and N9a, etc. [20,21] In Figure 1, the matrilineal component of Hui group was predominantly composed of haplogroups pertaining to East Asian-specific lineages (92.86%), while haplogroups U2, X2, T1, and HV, belonging to European-specific lineages, occupied the minority of lineages in Hui group (7.14%). The East Asian-specific haplogroups in Hui group mainly fell into macrohaplogroup N, which included haplogroups A (7.14%), N9 (3.06%), and Y1 (1.02%); into haplogroups F (15.31%), B (7.14%), and R* (5.10%), which belonged to major haplogroup R (a major branch of N; PhyloTree build 17, http://www.phylotree.org/index.htm); and into various clades of macrohaplogroup M, including haplogroups D4 (18.37%), C (10.20%), D5 (9.18%), M’ (8.16%), G (7.14%), and Z4 (1.02%). 

As depicted in Figure 1, the subclade D4 (18.37%) exhibited the highest proportion in Hui group. As the most typical branch of haplogroup D, the D4 was prevalent in modern populations from Northeast Asia [22], and also showed high frequencies in Han populations from North and Northeast China [23]. The D5 (9.18%) haplogroup was allocated to subclades D5a2, D5b1, and D5c + 16311 in Hui group, which was mainly found in East and Southeast Asia [24,25]. Haplogroup F (15.31%) in Hui group was mainly represented by the F1 (10.2%) subclade commonly found in Southeast Asia [26], as well as South and Southwest China [23]. The C7 (7.14%) haplogroup was dominantly presented in East Asia [27]. The B lineage (7.14%), including B4 and B5, was shown to be widespread among the North Asian [28] and Chinese Southern Han populations [23]. The haplogroup M7 (6.12%) was mainly found in East Asia and Southeast Asia [23,26,29]. It was also worth noting that the haplogroup G (7.14%) with its sub-haplogroup G2 was reported to exhibit high frequencies in Mongolic- or Turkic-speaking populations [30,31]. 

As for the European-specific lineages (U2, X2, T1, and HV) in the present Hui group, haplogroup U2 accounted for the majority (3.06%), which was a rare lineage spread across Central Asia, Europe, the Middle East, and North Africa [32]. In detail, the U2a and U2e branches discerned in this study were considered to be the subclades peculiar to South Asia [33,34] and Europe [35], respectively. 

### 3.3. Descriptive Statistical Indexes of Hui and Han Populations

The historical researches and previous studies implied that Hui group might have intimate relationships with Han populations from Chinese different regions [3]. Therefore, we simultaneously calculated the genetic diversities, neutrality tests, and mismatch distributions for Hui group and Han populations (CHB, CHS, MIN, and HAK) to further assess the population differences and commonness in a maternal genetic perspective. 

As shown in Table 1, in terms of Hui group, the overall diversity was considerably high with 97 different haplotypes, belonging to 83 different haplogroups, found in 98 individuals. Genetic diversity indexes, such as the haplotype diversity (Hd = 1.00), nucleotide diversity (pi = 0.00224), number of segregating sites (s = 640), and average number of pairwise nucleotide differences (k = 37.063), were also relatively informative. Moreover, the neutrality tests of Hui group presented significantly negative values, encompassing Tajima’s *D* (−2.39, *p* < 0.05) and Fu’s *F_S_* test (−24.09, *p* < 0.05). Mismatch distributions were also implemented to infer the history of Hui group with a unimodal pattern (Supplementary Figure S2), and the model test statistics, including SSD and HRI, were performed to evaluate whether a mismatch distribution was significantly divergent from the expected expansion model. Not surprisingly, the SSD (0.0015, *p* = 0.520) and HRI (0.0005, *p* = 0.989) in Hui group failed to significantly deviate from the model expectation of demographic expansion. For the selected references, four Han populations, containing CHB, CHS, MIN, and HAK, yielded significantly negative values for both Tajima’s *D* and Fu’s *F_S_* neutrality tests and no significant *p*-values for both SSD and HRI statistics, which was concordant with the neutrality test and mismatch distribution of Hui group. 

Briefly, the unequivocally significant departure of neutrality tests found in Hui group could be primarily explained by an excess of new and low-frequency mutations due to evolutionary forces, such as population expansion. The distribution curve of Hui group also supported the demographic history of sudden expansion with the unimodal mismatch distribution and non-significant model fit statistics (SSD and Hri). Four Han populations (CHB, CHS, MIN, and HAK) exhibited similar results of neutrality tests and unimodal mismatch distributions with Hui group, implying recent population expansions.

### 3.4. Genetic Divergence among Populations

#### 3.4.1. Analysis of Molecular Variance for Hui and 99 Worldwide Populations

It was reported that AMOVA could generate estimates of variance components, representing a correlation of haplotype diversities at different levels of hierarchical subdivision [36]. To some extent, AMOVA could further detect the existing factors that might have made sense for the formation of mtDNA diversity [23,37]. Therefore, we performed AMOVA by classifying 100 worldwide populations into various groups according to linguistic dialects and geographical regions (Table 2). Before grouping, the vast majority of variability was contributed to within-populations proportion as expected, 92.01% for the linguistic families and 92.08% for the geographic regions. The variation among populations within linguistic groups (5.57%) displayed a smaller proportion in contrast with that among populations within geographic groups (5.95%). After grouping, populations separated by linguistic families (2.42%) contained a higher percentage of variation in comparation with that of geographic groups (1.97%). In general, the high values of percentage of variation among groups would better explain the substructure of populations. The abovementioned results demonstrated that the linguistic grouping of complete mitogenome data from worldwide populations might provide a slightly better fit rather than geographic grouping. 

#### 3.4.2. Principal Component Analysis for Hui and 99 Worldwide Populations

**Figure 2:**
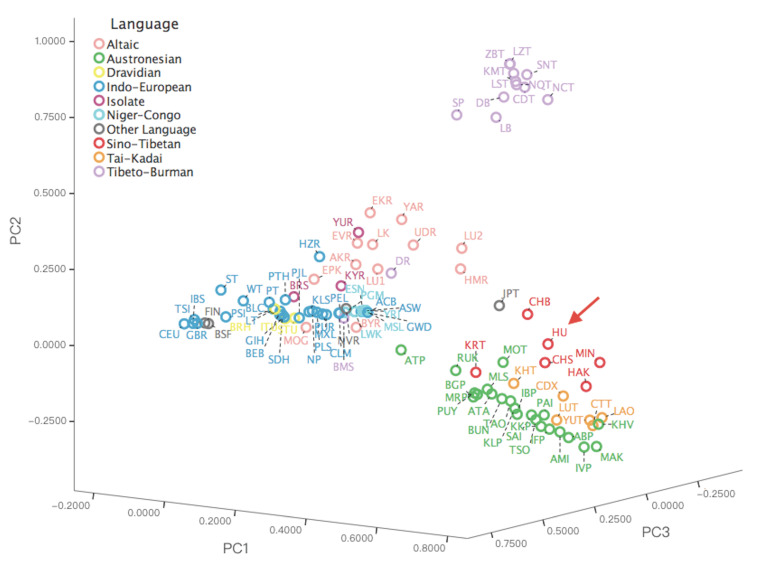
A principal component analysis (PCA) plot showing the genetic relationships between populations based on the haplogroup frequencies of 7372 complete mitochondrial sequences from 100 worldwide populations, the Hui group, and 99 reference populations. The red arrow refers to the studied Hui group (HU). The geographical origins of 100 populations in the plot are listed as follows: six populations from Africa, including ESN, GWD, LWK, MSL, YRI, and PGM; six from America, including ACB, PEL, MXL, CLM, PUR, and ASW; 16 from Central Asia, including LK, LU1, EPK, LU2, BRH, LTJ, PT, ST, WT, BLC, HZR, KLS, PTH, SDH, BRS, and HU; 29 from East Asia, including MOG, KHV, TSO, ATA, BUN, SAI, MAK, PAI, RUK, AMI, PUY, TAO, JPT, CHB, MIN, HAK, CHS, CDX, CDT, KMT, LZT, LST, NQT, DB, LB, NCT, SNT, SP, and ZBT; seven from Europe, including BSF, GBR, CEU, TSI, IBS, PLS, and FIN; 10 from North Asia, including UDR, EVR, EKR, YAR, AKR, BYR, HMR, NVR, KYR, and YUR; seven from South Asia, including ITU, STU, BEB, NP, GIH, PJL, and DR; 18 from Southeast Asia, including MOT, MLS, ABP, ATP, BGP, IBP, IFP, IVP, KKP, KLP, MRP, KRT, LAO, CTT, KHT, LUT, YUT, and BMS; and one from West Asia, PSI.

To estimate the genetic relationships between Hui group and 99 worldwide populations, PCA was implemented based on the haplogroup frequencies of 7372 complete mitochondrial sequences. A scree plot presenting the variance of each principal component was captured in Supplementary Figure S3. The first three principal components explained 28.5% of the total variation, in which the first, second, and third components accounted for 12.8%, 9.3%, and 6.4%, respectively. As shown in Figure 2, according to the AMOVA results, we divided 100 worldwide populations according to the linguistic families, which presented a higher population aggregation compared with that of geographic regions. All the populations marked by linguistic families were plotted in a world map, as shown in Supplementary Figure S4. In the PCA plot, part of the Sino-Tibetan-speaking groups in red were predominantly scattered in the East Asian cluster and the Hui group was primarily surrounded by Chinese Han populations, such as CHB and CHS. The Tibeto-Burman language in purple, one branch of Sino-Tibetan linguistic family, was mainly occupied by the Tibetan groups on top of the plot. The Tai-Kadai, the language once categorized into Sino-Tibetan linguistic family, was overshadowed by Southeast Asian populations scattered between Sino-Tibetan and Austronesian populations. The dispersal of Austronesian-speaking populations in East and Southeast Asia was plotted at the lower right corner of the plot with green color. The Altaic populations depicted in pink color were prevalently concentrated in Central and North Asia, distributing in the center of the plot. The Indo-European language was widely spoken by populations from America, Central Asia, Europe, and South Asia scattering on the left side of the plot in deep blue color. The African populations in light blue mainly belonged to the Niger–Congo family clustered between Indo-European and Altaic families. The remaining linguistic families were dispersed sporadically in the plot, including Dravidian, Uralic, Japanese-Ryukyuan, Isolate, Chukotko-Kamchatkan, and Basque languages.

Briefly, the above results hinted that Hui group might have intimate genetic relationships with Han populations, which might attribute to the phenomenon that they had more sharing haplogroups. In detail, several haplogroups predominately distributed in Hui minority such as D4 (18.37%), F1 (10.20%), D5 (9.18%), M7 (6.12%), and B4 (5.10%) were also detected to be widespread in Han populations [38]. The present results were concordant with the previously published studies. For example, the D4 haplogroup was reported to be widely distributed in Northern Han populations, whereas haplogroups F1, B4, and M7 were prevalent in Han populations from Southern China [23].

#### 3.4.3. Genetic Distance Analyses for Hui and 99 Worldwide Populations

**Figure 3:**
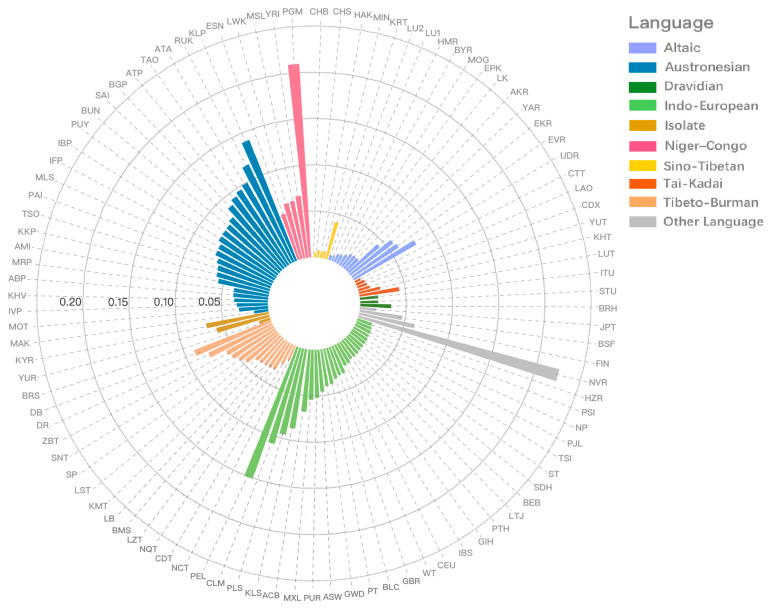
A circle histogram of pairwise *F_ST_* values between Hui and 99 worldwide populations based on the haplogroup frequencies.

To obtain a deeper understanding of Hui’s maternal origin, genetic distances (pairwise *F_ST_* values) were computed among the Hui group and 99 worldwide populations according to the haplogroup frequencies. Then, a circle histogram of pairwise *F_ST_* values was depicted to get a more intuitive insight of the genetic relationships between populations. According to the genetic differentiation criteria of Wright [39], genetic differentiations were defined as: *F_ST_* <0.05 for low differentiation, 0.05 < *F_ST_* < 0.15 for moderate differentiation, 0.15 < *F_ST_* < 0.25 for high differentiation, and *F_ST_* > 0.25 for very high differentiation [40]. The pairwise *F_ST_* values between Hui and other reference populations were ranged from 0.0052 to 0.2252 with the median value 0.0363. As shown in Figure 3, the reference populations were divided into different linguistic groups. The Hui group presented comparatively low deviations with populations speaking Sino-Tibetan, Tai-Kadai, and Altaic. Specifically, the Hui group was revealed to have smaller genetic divergences with Han populations, exhibiting the lowest degree with CHB (*F_ST_* = 0.0052), followed by MIN (*F_ST_* = 0.0073), HAK (*F_ST_* = 0.0073), and CHS (*F_ST_* = 0.0079). The Hui group also showed very low genetic distances (*F_ST_* < 0.01) with LU1, LU2, HMR, CTT, and LAO populations. In addition, the Hui group generally had relatively distant genetic relationships with the majority of Niger–Congolese- and Austronesian-speaking populations. Three populations from America (PEL = 0.1509), Africa (PGM = 0.2096), and North Asia (NVR = 0.2252) were identified as highly divergent populations with the Hui group, when the *F_ST_* value of 0.15 was set as the threshold.

#### 3.4.4. The Population Expansion Time of Hui and Han Populations

**Figure 4:**
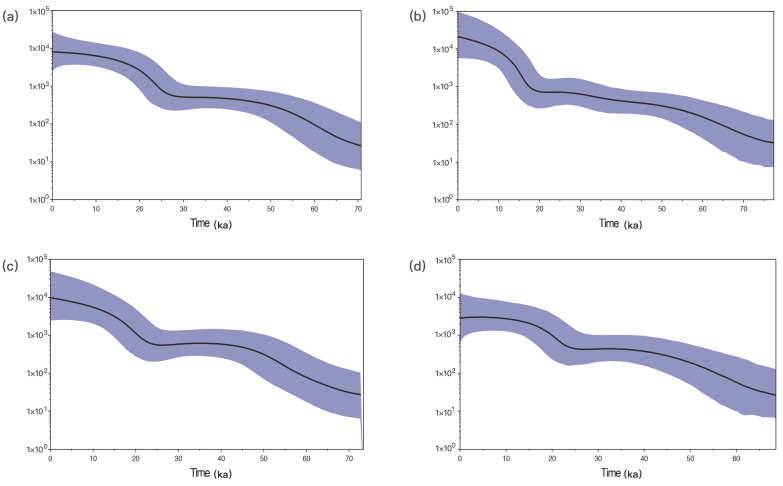
The Bayesian skyline plot (BSP) analyses are performed with the complete mitochondrial sequences to detect the demographic history of Hui (**a**), CHB (**b**), CHS (**c**), and MIN (**d**) populations. The *Y*-axis represents the assumed effective population size on a logscale. The black lines in bold represent the median population size. The blue line demarcates the boundary of the 95% highest posterior density.

As depicted above, the Hui group had relatively intimate relationships with Han populations. In addition, we further performed the Bayesian skyline plot (BSP) with the complete mitochondrial sequences of Hui group and Han populations to compare their population size dynamics. As presented in Figure 4a, BSP represented the median of the assumed effective population size of Hui group through time, and the population growth occurred since 25.71 (25.00–26.43) ka, a time period belonging to the Late Pleistocene. Similar population expansions could also be observed in CHS (Figure 4c) and MIN (Figure 4d) with population size growths at 20.75 (20.01–21.49) ka and 22.87 (22.18–23.56) ka, respectively, which were roughly consistent with the expansion of population along the Zhujiang river valley observed in the previous study [23]. The population expansion of CHB (Figure 4b), which might start at 16.43 (15.64–17.21) ka, was later than that of Hui group and two Han populations from South China. 

#### 3.4.5. Haplotypes Sharing between Hui and Worldwide Populations

To further explore the genetic sharing between the studied Hui group and reference populations around the world, we conducted extensively haplotypic comparisons of complete mitogenome of 7372 individuals and finally determined 1578 sequences intimate to Hui haplotypes to perform the median networks. Given the limited size and the widespread haplotype distributions of sampled Hui individuals, we had to take all the haplotypes from the Hui group into account, and according to the network results, the reference individuals within one mutation difference with Hui haplotypes were simply counted. 

**Figure 5:**
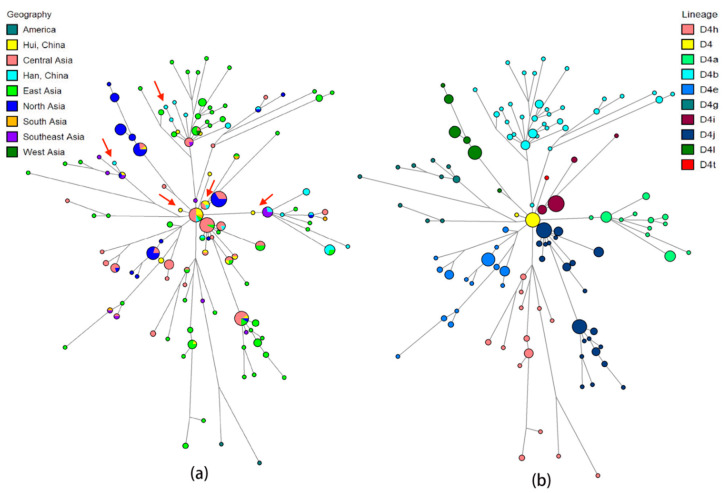
The median network of mitochondrial D4 haplogroup. The left plot (**a**) colored by geographic origins, and the right plot (**b**) colored by haplogroups. The size of the nodes is proportional to the number of individuals carrying that node. The length of the branch is positively correlated with the number of different mutations, the longer the branch, the more different the mutations.

**Figure 6:**
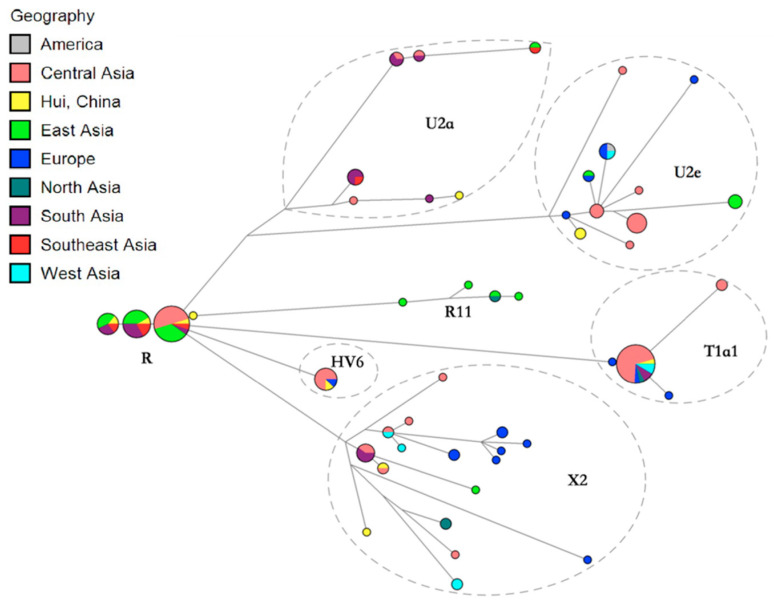
The median network of European-specific lineages appearing in Hui group coupled with reference individuals from worldwide populations. The size of the nodes is proportional to the number of individuals carrying that node. The length of the branch is positively correlated with the number of different mutations, the longer the branch, the more different the mutations.

For the East Asia-specific haplogroups, Hui individuals predominantly clustered with individuals from Han, Uygur, and Tibetan populations. In detail, Hui individuals displaying the same haplotypes with Han individuals or differing from Han individuals by only one mutation accounted for 45.92%, reflecting close genetic relatedness between Hui and Han individuals. These Hui individuals fell within 35 different haplotypes, including haplotypes belonging to macrohaplogroup M (D4, C7, C4d, D5, M7, G2b, and G1c) and to macrohaplogroup N (F1, B4, A15, and N9). For example, the median network of mtDNA haplogroup D4, the most typical linage in Hui group, was presented in Figure 5. Plots 5a and 5b were totally identical, but marked by different criteria with geographical origins and haplotype lineages, respectively. As a result, Hui and Han individuals tend to share the same haplotypes or differ by one mutation on the D4 haplogroup and four sub-haplogroups D4a, D4b, D4i and D4g, as indicated by the red arrow in the plot. For other reference populations, a total of 36.73% Hui individuals shared haplotypes with Uygurs, represented by 30 haplotypes, which were classified to major M lineage (D4, D5b, C4a, G, and M7) and N lineage (F1, B4c, A, and N9). However, the large sample size of Uygur population, over 700 samples, should not be ignored. The sharing of the same haplotypes or haplotypes with one mutation difference was also observed between Hui (21.43% of the total number) and Tibetan individuals, which mainly emerged on the haplogroups F1, D4, D5a, and A. About 6.12% and 9.18% of Hui minority tended to share the same branches with Mongolian related groups (such as BYR and HMR) and Xinjiang Tajik group, respectively. Only two Hui individuals were clustered with Persians scattered on haplogroup D4b. Some Hui individuals were also observed to share branches with individuals from South and Southeast Asia, which were not described in detail here. The networks of East Asia-specific haplogroups except the D4 haplogroup were described in Supplementary Figure S5. With respect to European-specific lineages (Figure 6), two Hui individuals that exhibited the identical U2e haplotype were clustered together with an European individual within one mutation difference. The sequences belonging to the HV6 haplogroup were found in one Hui individual, one European, and six Central Asians. Furthermore, one Hui individual carrying the T1a1 haplotype was grouped with one European, one Mongolian, two Persians, and 17 Central Asians.

From the perspective of historical development, it was recorded that the Hui group were of varied ancestries, many of whom descended from Central Asians and West Asians, such as Persians, Arabs, and Turks who intermarried with the local Han individuals [3,4,41]. It was said that their intermarriage was largely promoted by a historically political factor during the medieval Chinese dynasties, especially during the Yuan dynasty. In addition, the economic factor also played a certain role in the formation of Hui minority. For example, the economic exchanges along the Silk Road might simultaneously facilitate the population integrations [4]. Finally, partial Uygur, Mongolian, and Tibetan people might also contribute to the eventual formation of the Hui minority according to the record [4].

The result of median networks hinted that the maternal genetic components of Hui minority might be relatively abundant and complex. Initially, the extensive gene components from the Han population might have been assimilated into the Hui group during the formation process. The present notions were further supported by the previously published researches. For example, Xie et al. investigated the genetic and forensic characteristics of nine different geographical Hui groups using 157 Y-SNPs and 27 Y-STRs and finally found that Hui groups from the Northwest China, Sichuan, and Shandong provinces displayed close genetic affinities with Chinese Han [42]. He et al. and Zhou et al. also found that there was an intimate genetic relationship between the Ningxia Hui group and Chinese Han population with a significant East Asian ancestry component [43,44]. Yao et al. presented that a significant genetic homogeneity was found between Linxia Hui and Han [45]. The close relationships between the Hui and Han populations were also confirmed in our previous researches [46,47,48,49]. Furthermore, we also found some clues for the genetic affinity between the Hui and Tibetan groups, which was reported arising from a mixture of multiple ancestral gene pools with East Asian, Central Asian, and South Asian components [2,50]. This affinity might be partially attributed to the intermarriage between the Huis and Tibetans as early as the 17th century [51]. For Central Asian populations, a substantial gene flow possibly emerged between Hui and Xinjiang Uygur, all of whom shared the same religious belief and located along the Silk Road [3,4]. Yu et al. supported this opinion based on the research of mitochondrial DNA polymorphisms in Chinese Han, Uygur, Kazak, and Hui ethnic groups [52]. Further, Hui group might have minor genetic exchanges with the Xinjiang Tajik group who was conceived as the descendants of the remaining Eastern Iranians that resided along the Silk Road with Islamic faith [53]. According to the historical records, the Hui individuals were once ruled by the Mongol Yuan dynasty. Hong et al. suggested the relatively close relationship between Hui and Mongolian groups [2]. However, our results did not show much evidence of genetic exchange between Hui and Mongolian groups, which might be ascribed to the limited resources of existing complete mtDNA genome data of Mongolians with merely 20 individuals in this study. Although it was documented that the Hui might be partially descended from the Persians, this study found little genetic proximity between modern Persians and the present Hui individuals. Eventually, four Hui individuals belonging to haplogroups U2e, HV6, and T1a1 were clustered with Europeans, which might largely be attributed to the gene flow from Central or West Asians during the formation of Hui group [3,4,41].

## 4. Conclusions

The present study provided the first set of complete mitochondrial genome data of 98 Hui individuals residing in Northwest China. According to the present research, most of the mtDNA haplogroups of Hui group were classified into the lineages peculiar to East Asia, while a few haplogroups into the lineages peculiar to Europe. This notion might imply a dominant contribution of East Eurasian to Hui’s maternal gene pool and a concomitant dilution of its West Eurasian provenance. In detail, the Hui group exhibited greater genetic affinities with Chinese Han populations, largely ascribing to the widespread haplogroups D4, D5, M7, B4, and F1 in these populations. Further, the expansion time of Hui group was during the Late Pleistocene. Compared with Northern Han, the Hui group tended to display a closer expansion time with Southern Han populations. Finally, we also found that the present Hui group might contain different degrees of genetic imprints from the Uygur, Tibetan, and Tajik groups, suggesting the existence of multi-ethnic integration events in the process of forming the maternal genetic landscape of Hui group. Overall, due to the genetic complexity of Hui group in China, more Hui samples from different regions need to be taken into consideration to obtain a more comprehensive sight of its maternal genetic compositions. 

## Figures and Tables

**Table 1:** Diversity indexes and neutrality tests for the studied Hui population and interested populations based on a complete mitogenome.

				Genetic Diversities				Neutrality Tests		Mismatch Distributions	
Population	Abbreviation	n	h	Hd (SD)	S (Eta)	k	Pi (SD)	Tajima’s *D* (*p*)	Fu’s *Fs* (*p*)	SSD (*p*)	HRI (*p*)
Hui in Northwest China	HU	98	97	1.000(0.002)	640(650)	37.06	0.0022(0.00006)	−2.39 (0.000)	−24.09 (0.001)	0.0015 (0.520)	0.0005 (0.989)
Beijing Han Chinese	CHB	85	85	1.000(0.002)	614(619)	38.37	0.0023(0.00005)	−2.37 (0.000)	−24.15 (0.001)	0.0029 (0.429)	0.0007 (0.999)
Southern Han Chinese	CHS	50	50	1.000(0.004)	442(445)	38.36	0.0023(0.00007)	−2.23 (0.001)	−21.91 (0.000)	0.0021 (0.652)	0.0015 (0.981)
Minnan Han	MIN	50	48	0.998(0.004)	397(400)	36.09	0.0022(0.00006)	−2.16 (0.003)	−17.98 (0.000)	0.0013 (0.744)	0.0014 (0.983)
Hakka Han	HAK	45	39	0.994(0.006)	369(372)	34.73	0.0021(0.00007)	−2.16 (0.002)	−7.38 (0.035)	0.0022 (0.510)	0.0046 (0.171)

n: Number of sequences; h: Number of haplotypes; Hd: Haplotype diversity (standard deviation); S: Number of segregating sites (total number of mutations); k: Average number of pairwise nucleotide differences; Pi: Nucleotide diversity; SSD: Sum of squared deviations; HRI: Harpending’s raggedness index.

**Table 2:** Analysis of molecular variance (AMOVA) results based on different groups for worldwide populations.

			Percentage of Variation		
Groupings	Number of Populations	Number of Groups	Within Populations	Among Populations with in Groups	Among Groups
Linguistic families of worldwide populations	100	13	92.01	5.57	2.42
Geographic distributions of worldwide populations	100	9	92.08	5.95	1.97

## Supplementary Materials

The following are available online at https://www.mdpi.com/2073-4425/11/11/1352/s1. Figure S1: The sequencing performance of the MultipSeqTm AImumiCap mtDNA Whole Genome Panel assessed from the read depth of 98 Hui individuals; Figure S2: Mismatch distribution of Hui group with a unimodal pattern; Figure S3: A scree plot represented the variance of each principal component captured; Figure S4: A world map plotted all the populations in this study and was colored by language families; Figure S5: The networks of East Asia-specific haplogroups except the D4 haplogroup; Table S1: The comparison populations and the corresponding references involved in this study; Table S2: Variants and haplogroup information of the 98 Hui individuals.
