# Supplementary figures and images for: The Genetic Structure of Chinese Hui Ethnic Group Revealed by Complete Mitochondrial Genome Analyses Using Massively Parallel Sequencing
Source: Genes (Basel). 2020 Nov 14;11(11):1352. doi: 10.3390/genes11111352 (PMC7698084; doi:10.3390/genes11111352)

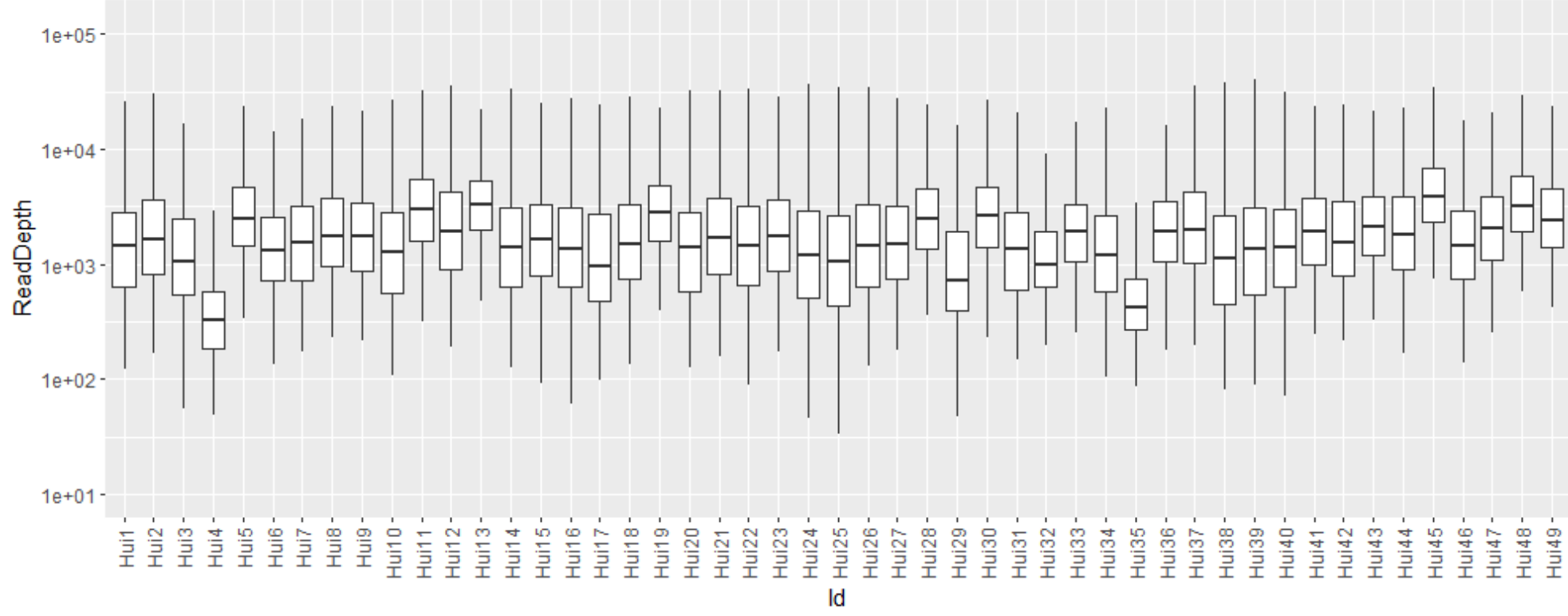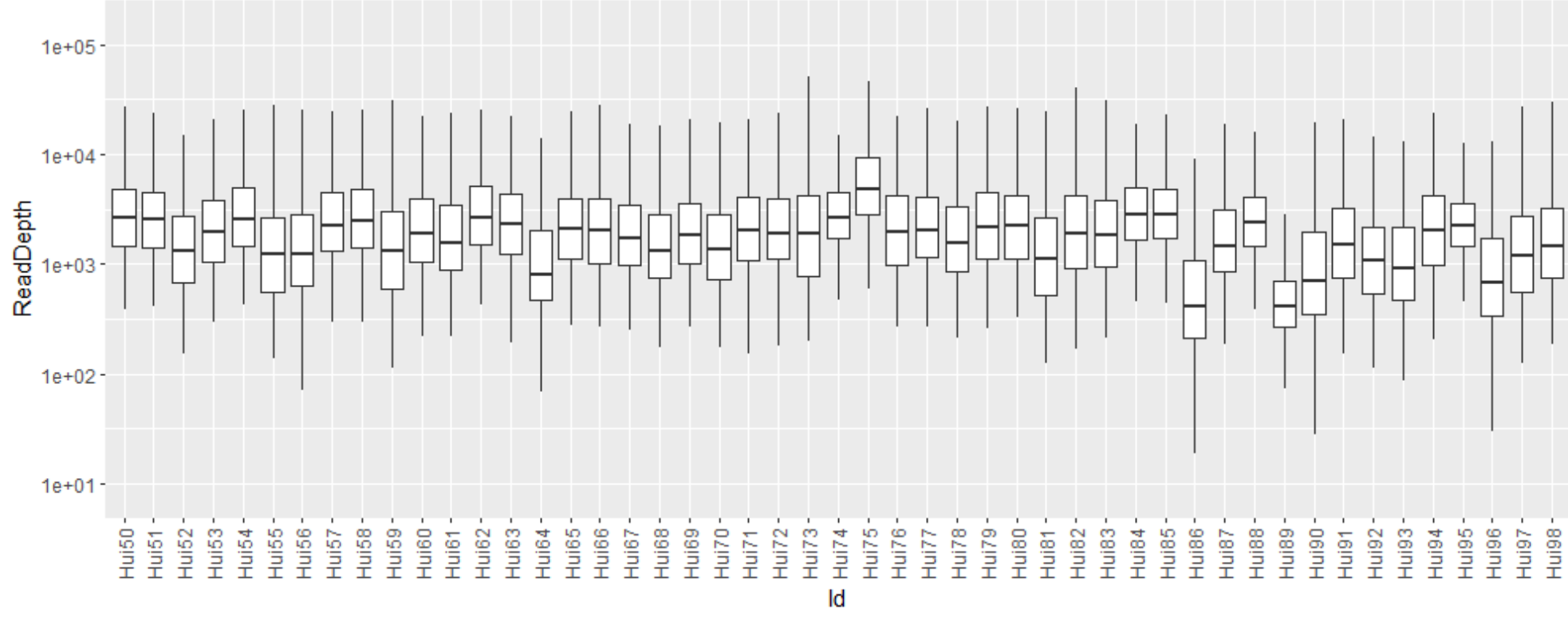

Supplement: Supplementary file 1 [file genes-11-01352-s001.zip › Supplementary Files /Supplementary Figure 1.pdf]

# Mismatch distribution of Hui population

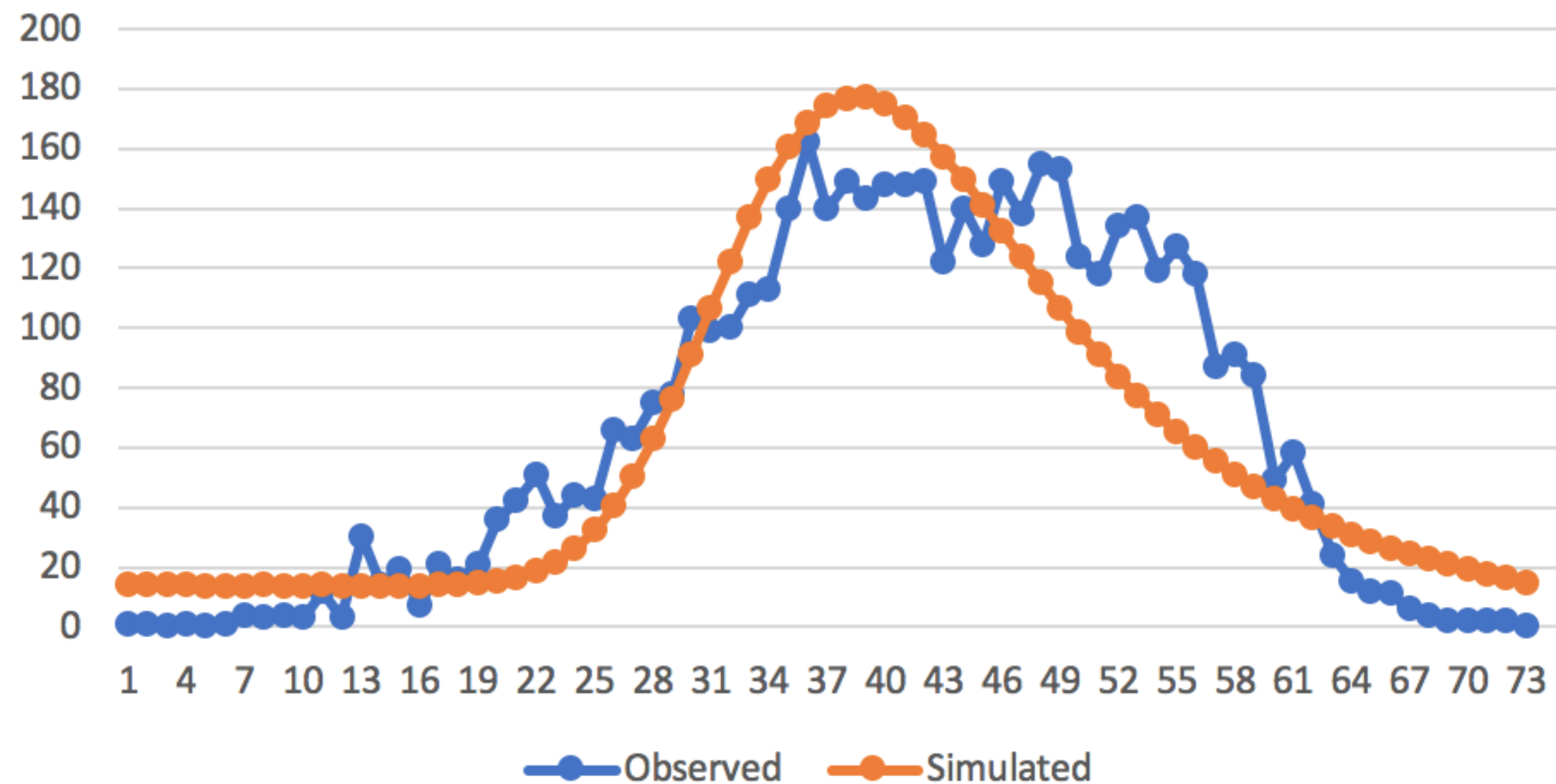

Supplement: Supplementary file 1 [file genes-11-01352-s001.zip › Supplementary Files /Supplementary Figure 2.pdf]

# Scree Plot

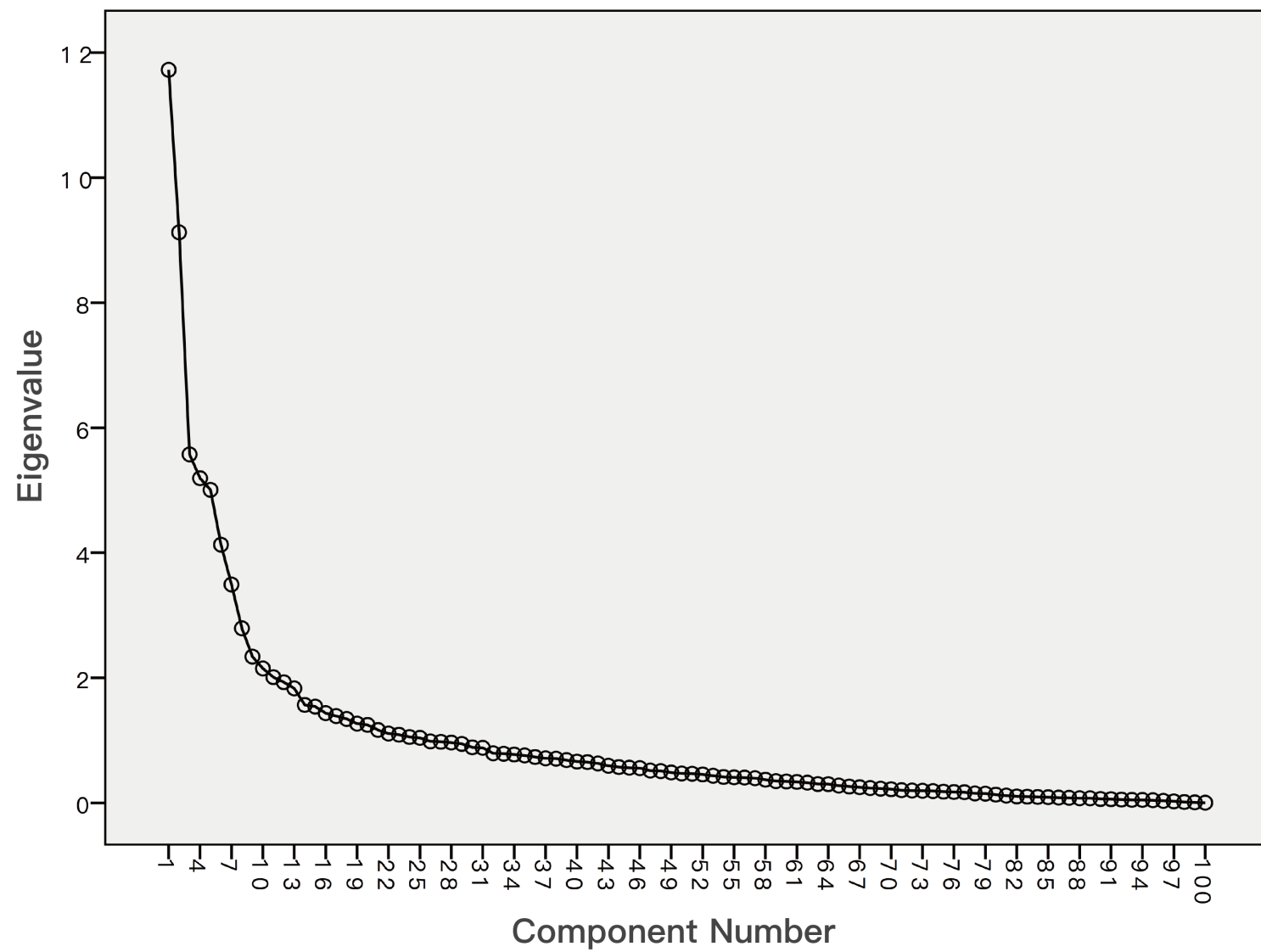

Supplement: Supplementary file 1 [file genes-11-01352-s001.zip › Supplementary Files /Supplementary Figure 3.pdf]

Latitude

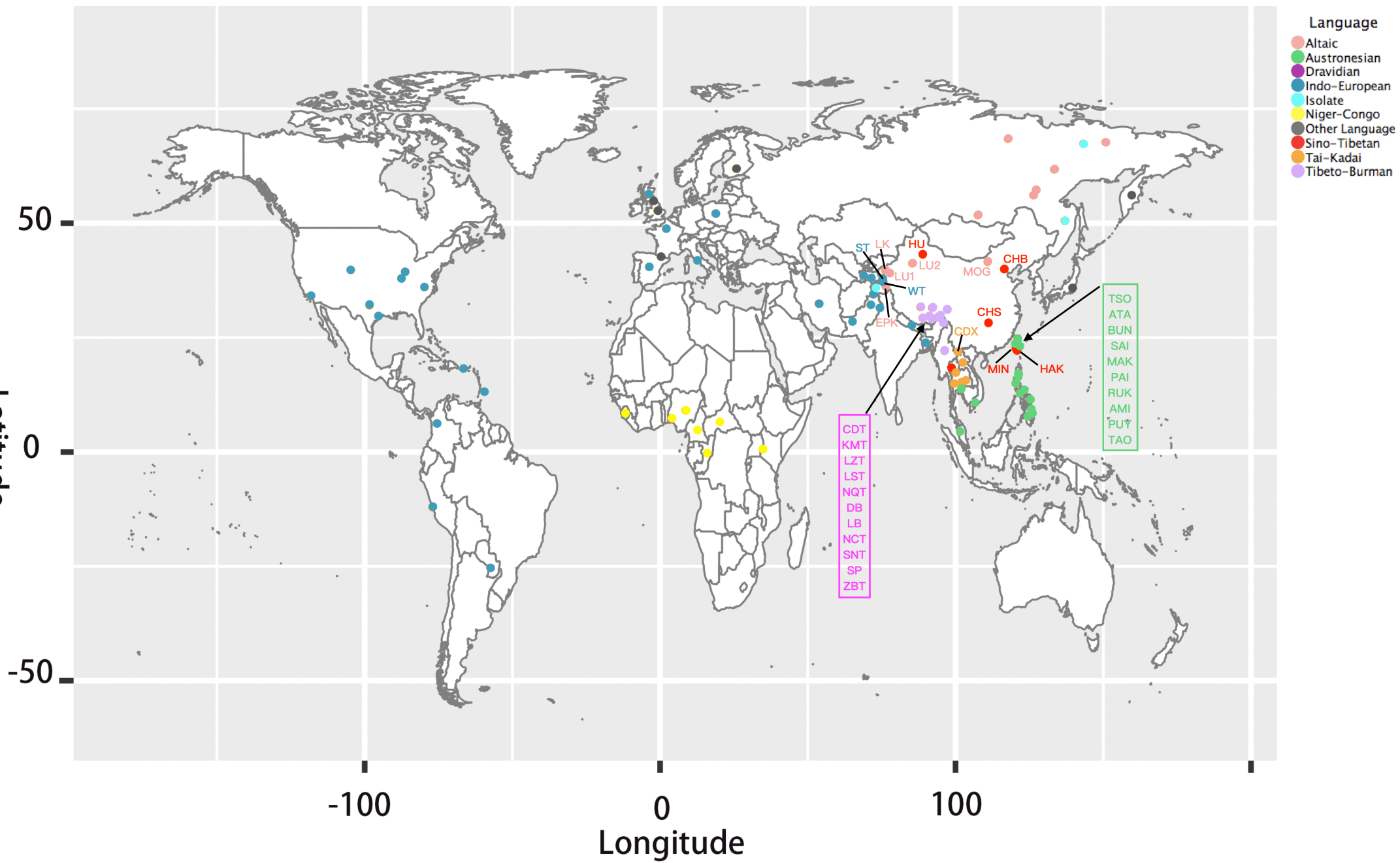

Supplement: Supplementary file 1 [file genes-11-01352-s001.zip › Supplementary Files /Supplementary Figure 4.pdf]

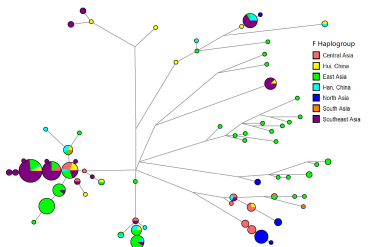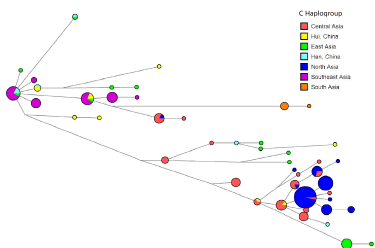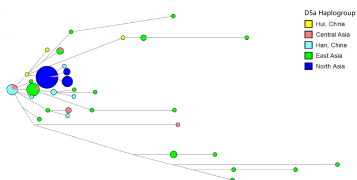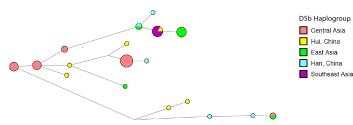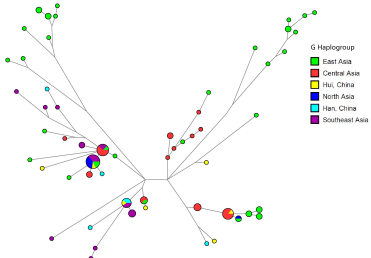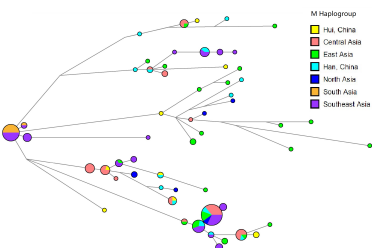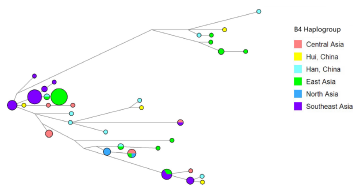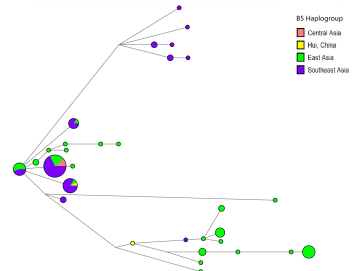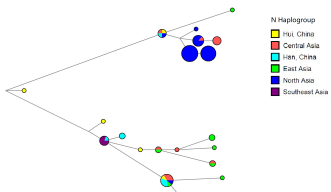

Supplement: Supplementary file 1 [file genes-11-01352-s001.zip › Supplementary Files /Supplementary Figure 5.pdf]
